# Transient Antibiotic Tolerance Triggered by Nutrient Shifts From Gluconeogenic Carbon Sources to Fatty Acid

**DOI:** 10.3389/fmicb.2022.854272

**Published:** 2022-03-11

**Authors:** Christopher J. Hartline, Ruixue Zhang, Fuzhong Zhang

**Affiliations:** ^1^Department of Energy, Environmental and Chemical Engineering, Washington University in St. Louis, Saint Louis, MO, United States; ^2^Division of Biology and Biomedical Sciences, Washington University in St. Louis, Saint Louis, MO, United States; ^3^Institute of Materials Science and Engineering, Washington University in St. Louis, Saint Louis, MO, United States

**Keywords:** antibiotic tolerance, persistence, nutrient shift, bacterial metabolism, metabolic positive feedback, fatty acid pathway

## Abstract

Nutrient shifts from glycolytic-to-gluconeogenic carbon sources can create large sub-populations of extremely antibiotic tolerant bacteria, called persisters. Positive feedback in *Escherichia coli* central metabolism was believed to play a key role in the formation of persister cells. To examine whether positive feedback in nutrient transport can also support high persistence to β-lactams, we performed nutrient shifts for *E. coli* from gluconeogenic carbon sources to fatty acid (FA). We observed tri-phasic antibiotic killing kinetics characterized by a transient period of high antibiotic tolerance, followed by rapid killing then a slower persister-killing phase. The duration of transient tolerance (3–44 h) varies with pre-shift carbon source and correlates strongly with the time needed to accumulate the FA degradation enzyme FadD after the shift. Additionally, FadD accumulation time and thus transient tolerance time can be reduced by induction of the glyoxylate bypass prior to switching, highlighting that two interacting feedback loops simultaneously control the length of transient tolerance. Our results demonstrate that nutrient switches along with positive feedback are not sufficient to trigger persistence in a majority of the population but instead triggers only a temporary tolerance. Additionally, our results demonstrate that the pre-shift metabolic state determines the duration of transient tolerance and that supplying glyoxylate can facilitate antibiotic killing of bacteria.

## Introduction

Antibiotic tolerance describes the ability of bacteria to survive longer periods of antibiotic treatment while remaining genetically susceptible to antibiotic killing (Meylan et al., 2018). Heterotolerance, or persistence, is a special case of antibiotic tolerance where only a sub-population of an isogenic culture displays antibiotic tolerance, leading to a bi-phasic antibiotic killing kinetics—a rapid killing phase for the susceptible sub-population followed by a slow killing phase for the tolerant population. Tolerance and persistence have been suspected as an important cause of recurrent and recalcitrant bacterial infections, particularly when the disease-causing bacterium appears to remain susceptible to antibiotic killing during *in vitro* assays (Ojha et al., 2008; Mulcahy et al., 2010; Fauvart et al., 2011; Morrison et al., 2020). Further, tolerance can promote the ability of bacteria to acquire antibiotic resistance (Levin-Reisman et al., 2017; Windels et al., 2019; Liu et al., 2020), which reduces antibiotic efficacy in clinical settings and is contributing to an impending global public health crisis (Aslam et al., 2018; Talebi Bezmin Abadi et al., 2019). These public health problems have led to increased interest in understanding antibiotic tolerance mechanisms in microbes.

The degree of metabolic activity has been deeply explored as a central feature of antibiotic tolerance and persistence (Shah et al., 2006; Amato et al., 2014; Lopatkin et al., 2019). Increased antibiotic tolerance is observed in metabolic environments where cells are slowly growing or non-growing, including in stationary phase and biofilms (Gilbert et al., 1990; Jõers et al., 2010; Orman and Brynildsen, 2015). Genomic screens for antibiotic tolerance genes have identified several mutations in metabolic pathways which vary the frequency of antibiotic tolerant cells (De Groote et al., 2009; Bernier et al., 2013). These mutations affecting metabolic genes may contribute to antibiotic resistance under repeated antibiotic selection (Zampieri et al., 2017; Lopatkin et al., 2021). Additionally, antibiotic tolerant cells were shown to have lower levels of ATP (Shan et al., 2017; Manuse et al., 2021), increased levels of alarmones (Hauryliuk et al., 2015; Svenningsen et al., 2019), and reduced translation (Gefen et al., 2008). These metabolic mechanisms alter the efficacy of antibiotics, leading to the prolonged survival of antibiotic tolerant cells (Stokes et al., 2019). Persistence has an additional requirement of maintaining a sub-population of tolerant cells while the remainder of the population is antibiotic susceptible (Balaban et al., 2019). Thus, several stochastic mechanisms for generating and maintaining low metabolic activity, particularly stochastic induction of toxin-antitoxin pairs and stochastic accumulation of (p)ppGpp, have been explored in connection with persistence (Balaban, 2004; Germain et al., 2015; Schmitz et al., 2017; Svenningsen et al., 2019; Evans and Zhang, 2020).

Nutrient shifts are one of the mechanisms that have been shown to produce increased sub-populations of antibiotic tolerant cells (Radzikowski et al., 2017). During a nutrient shift, bacteria need to adjust their metabolic activities for different nutrient sources. The ability to generate a tolerant sub-population during metabolic adjustment may have evolutionary benefits to the entire population (Van Boxtel et al., 2017). Diauxic shifts from glucose to fumarate, glycerol, and succinate have been shown to result in bi-phasic killing kinetics with elevated levels of *Escherichia coli* persisters to both ofloxacin (Amato et al., 2013; Amato and Brynildsen, 2014) and ampicillin (Amato and Brynildsen, 2015) antibiotics. Similarly, complete shifts from glucose to fumarate resulted in an apparent mono-phasic killing kinetics with a large population of extremely slow-growing *E. coli* cells that were tolerant to many antibiotics with diverse mechanisms of action (Radzikowski et al., 2016), while only a small fraction of cells were able to resume growth on fumarate (Kotte et al., 2014). It was proposed that bistability in a positive feedback loop involving a phosphoenolpyruvate (PEP) flux sensor and the enzyme fructose-1,6-bisphosphatase (Fbp) was responsible for creating a two-population response to nutrient shifts. Positive feedback has been highlighted for its ability to increase cell-to-cell variability in gene networks and produce bistability, and can be an important mechanism in maintaining cells in the persister state (Eldar and Elowitz, 2010). Many metabolic regulatory networks are also controlled by positive feedback loops, particularly in the uptake of carbon sources such as carbohydrates (e.g., lactose, arabinose, xylose, and glycerol) and fatty acids (FAs) from the environment (Weissenborn et al., 1992; Song and Park, 1997; Cronan and Subrahmanyam, 1998; Ferrández et al., 2000; Ozbudak et al., 2004; Megerle et al., 2008; Hartline et al., 2020). However, it is not known whether the prevalent feedback loops in nutrient uptake can commonly lead to elevated levels of persistence during nutrient shifts.

In this work, we studied *E. coli* nutrient transition to FA because both FA transport and catabolic pathways are regulated by a positive feedback loop. Recent work in pathogenic bacteria has highlighted that utilization of exogenous FAs from the host environment plays a central role in regulating virulence factors in a broad-range of Gram-negative pathogens, including *E. coli* (Pifer et al., 2018; Pan et al., 2020; Ellermann et al., 2021). Thus, nutrient transitions to FA catabolism are associated with increased pathogenicity, but these transitions remain an understudied mechanism with respect to its effect on antibiotic tolerance. To avoid complication from the Fbp regulatory loop that may form bistability (Kotte et al., 2014), we focus on shifts from gluconeogenic carbon sources such as glycerol. Because both glycerol and FA require Fbp for growth (Fraenkel and Horecker, 1965), switching between gluconeogenic carbon sources should avoid triggering major changes in the activity of the Fbp loop. Distinct from the mono-phasic and bi-phasic killing kinetics previously reported during glycolytic-to-gluconeogenic switches, we observed a transient tolerance phase, where the population displays nearly universal tolerance to ampicillin during the first 8 h right after glycerol-to-FA shift. The transient tolerant phase was followed by a rapid killing phase for 98% of cells, followed by a persister phase with slower killing kinetics. This three-phase killing kinetics was observed when switching from at least five different gluconeogenic carbon sources (i.e., glycerol, pyruvate, malate, succinate, and acetate) tested in this study. We genetically fused the FA transport gene *fadD* (encodes the acyl-CoA ligase FadD) to a yellow fluorescent protein (YFP) and tracked transport expression kinetics after the nutrient shift. The results showed that the period of transient tolerance correlates well (*R*^2^ = 0.82 in the absence of glyoxylate and *R*^2^ = 0.98 in the presence of glyoxylate) with the time needed for FadD to accumulate to a threshold before resuming growth. We demonstrate that the activity of the positive feedback loop in the glyoxylate bypass modulates the timing of both transient tolerance and FadD production on shifts to FA. These results demonstrate a fundamental difference in *E. coli* response to gluconeogenic-to-FA nutrient shifts compared to previously reported glycolytic-to-gluconeogenic switches. Overall, our results have broad implications for the relation between metabolic regulations, nutrient shifts, and β-lactam antibiotic tolerance.

## Materials and Methods

### Strains, Plasmids, and Construction

All strains were derived from *E. coli* NCM3722, which was obtained from the Coli Genetic Stock Center (Yale, United States). All strains, plasmids, and primers used are given in Supplementary Table S2 and the plasmid sequences are given in Supplementary Table S3. Phusion DNA polymerase, restriction enzymes, and T4 ligase used in plasmid construction were purchased from Thermo Fisher Scientific (Waltham, MA, United States). Primers were synthesized by Integrated DNA Technologies (Coralville, IA, United States).

#### Acyl-CoA Biosensor Strain

The Acyl-coA biosensor plasmid pSARk-yemGFP was constructed from three parts following standard enzyme digestion and ligation protocols. The bglBrick vector pS5k-rfp (Lee et al., 2011) was digested by *AatII* and *XhoI*. The pAR promoter was obtained by digesting pBARk-rfp (Xiao et al., 2016) with *AatII* and *BglII*. The yemGFP sequence was amplified by PCR yemGFP_F and yemGFP_R (Supplementary Table S1) and digested with *BglII* and *XhoI*. Parts were ligated and transformed into *E. coli* NCM3722 to make the Acyl-coA Biosensor Strain.

#### FadD-YFP Strain

The FadD-YFP strain was constructed by following the pTarget-pCas homologous recombination system protocol as described previously (Jiang et al., 2015, 2017). Homology arms with 200–300 base pairs upstream and downstream of FadD were amplified from *E. coli* NCM3722 genomic DNA by PCR. The YFP gene was amplified from a codon optimized plasmid (Cox et al., 2010). The *fadD* and YFP genes were separated by a flexible glycine-serine-rich linker constructed on primers (Bai et al., 2019). A guide RNA with the following sequence was synthesized on primers and amplified along with the pTarget backbone: TGACGACTGACTTAACGCTC. PCR products were then assembled *via* Golden Gate Cloning to form pTargetF-FadD-YFP. The pTarget plasmid was then used to integrate YFP into the genome of *E. coli* NCM3722 transformed with pCas. Genome integration was verified by colony PCR and sequencing, and the genome integrated strain was cured of the pTarget and pCas plasmids. The final sequence of the FadD-YFP strain in the genome region of *fadD* is given in Supplementary Table S3.

### Growth Media

Cell growth and nutrient shift experiments were performed in M9 minimal media with corresponding carbon source [M9 minimal salts supplemented with 75 mM MOPS at pH 7.4, 2 mM magnesium sulfate, 1 mg/L thiamine hydrochloride, 10 μM iron(II) sulfate, 100 μM calcium chloride, 3 μM ammonium heptamolybdate, 0.4 mM boric acid, 30 μM cobalt(II) chloride, 15 μM copper(II) chloride, 80 μM manganese(II) chloride, and 10 μM zinc sulfate]. Media was supplemented with sodium salts of each carbon source at a concentration of 72 mM carbon: 4 mM oleate, 36 mM acetate, 24 mM pyruvate, 24 mM glycerol, 18 mM (S)-malate, and 18 mM succinate. For experiments with co-utilization of glycerol and FA, the ratios of glycerol and oleate were adjusted to maintain a total of 72 mM carbon atoms in the media. For experiments with co-utilization of carbon and glyoxylate, 9 mM glyoxylate was used in all conditions, along with following concentrations of each carbon source such that 72 mM of carbon atoms was maintained: 3 mM oleate, 24 mM acetate, 18 mM pyruvate, 18 mM glycerol, 13.5 mM (S)-malate, and 13.5 mM succinate. All cultures were supplemented with appropriate antibiotic for selection (Ampicillin, 100 μg/ml; Kanamycin, 50 μg/ml).

### Acyl-CoA Biosensor Activity

Single colonies of the acyl-CoA biosensor strain were grown in 3 ml LB media for 2–3 h, then washed twice in M9 without a carbon source. Cultures were shifted into 3 ml M9 media supplemented with specified glycerol and oleate concentration and grown for an additional 3–5 h. Cells were again washed twice in M9 without a carbon source and were transferred M9 media supplemented with the specified glycerol/oleate ratios in a 96-well plate. Cells were diluted to a density of ~2 cells/μl so that the glycerol/oleate ratio does not significantly change during cell growth. Cells were grown for 9 h; then, growth was halted by addition of 100 μg/ml of rifampicin and incubated on ice for at least 15 min prior to measurement. Samples were analyzed with a Guava easyCyte HT5 flow cytometer (Luminex Corporation, Austin, TX, United States) with blue 488 nm excitation laser and the green 525 nm emission filter.

### Nutrient Shifting and Colony Counting Assays

All nutrient shifts were performed following previous methods (Radzikowski et al., 2016). Specifically, cells were cultivated in a pre-shift medium at 37°C and were kept in exponential growth phase for 14–17 h. Cells were then collected and centrifuged in a pre-chilled centrifuge (4°C) at 4,500 rcf for 10 min. Supernatant was discarded and cells were resuspended in chilled M9 without carbon source. Three washes were performed. Finally, cell density (OD_600_) was normalized to 0.5 in M9 without carbon, and then diluted 1:5 into pre-warmed oleate media so that the final cell density was 0.1, and the final oleate and ampicillin concentrations were 4 mM and 100 μg/ml, respectively, and with a final culture volume of 25 ml. At times indicated, 1 ml culture was transferred to centrifuge tubes pre-filled with 200 μl of phosphate buffered saline (PBS, pH 7.4). Cells are centrifuged at 4,500 rcf, 4°C, and washed four times in PBS. Finally, cell pellets were resuspended to a final volume of 1 ml, and serial dilutions were also performed in PBS. For each diluted culture, 10 μl was transferred into LB agar plates and incubated for 12 h at 37°C. Colonies were counted to determine the colony forming units per ml of culture (CFU/ml).

### Transient Tolerance Time Determination

To quantify the length of time where the cells are transiently tolerant to ampicillin, we fit the colony counting data to a modified two-population Gompertz model, which has been widely used to describe kinetics of bacterial growth (Zwietering et al., 1990) and mortality (Kirkwood, 2015):


Survival=(1−p)×1−exp−exp1+μTT×e×(t−τTT) +(p)×1−exp−exp1+μPER×e×(t−τPER)

where *p* is the persister fraction, $\mu_{TT}$ and $\mu_{PER}$ are the maximum killing rates of the transient tolerant and persister population, respectively, and $\tau_{TT}$ and $\tau_{PER}$ are the transient tolerance times of the transient tolerant and persister fractions, respectively. Next, we assume that the tolerance time of the transient tolerant fraction and persister fraction are equal, i.e., $\tau_{TT}=\tau_{PER}=\tau .$ Thus, only the maximum rate of killing distinguishes the transient tolerant and persister populations.

To fit the data, CFU/ml counts were first normalized to the CFU/ml from time point zero and converted to a log_10_ scale. All data from replicates of each shifting experiment were fit to the two-population Gompertz model using the MATLAB R2020B curve fitting tool (cftool). An initial transient tolerance time was determined; the data were then re-normalized to the average CFU/ml counts prior to the tolerance time for each replicate. The re-normalized data were fit again, and the parameters along with their 95% CIs and SEM were determined.

### FadD-YFP Kinetic Assays

Cells were grown in different pre-shift carbon sources at steady state as described for Colony Counting Assays. Cells were centrifuged and washed in M9 without carbon source for four times. Washed cells were then transferred to a Falcon 96-Well Imaging Microplate (Corning, NY, United States) and diluted to an initial OD_600_ of 0.08 in oleate media with or without ampicillin, with a final volume of 150 μl. An Infinite F200PRO plate reader (TECAN, Männedorf, Switzerland) was used to make automated OD_600_ and fluorescence measurements (Excitation: 514 nm, Emission: 552 nm) every 6 min with constant shaking and 37°C temperature control. Fluorescence measurements are normalized by OD_600_ to give an estimate of FadD concentration.

### Lag Phase, Threshold, and Accumulation Time Calculations

Lag phase was determined by a similar protocol as described previously (Basan et al., 2020). The steady state growth rate after nutrient shift was first calculated by fitting a line to the natural log of the OD_600_ for the first hour after the culture density increases 8-fold from its initial density. The time point where this line intersects the initial culture density is the end of the lag phase. The fluorescence measurement at the closest measured time point is used as YFP/OD_600_ at the lag time. The FadD threshold was determined by taking the average YFP/OD level at the end of the lag phase across all nutrient shift conditions without glyoxylate. The accumulation time was determined as the first time point where the YFP/OD_600_ was above this threshold and the YFP/OD_600_ at all subsequent time points also exceeded the threshold.

### Tolerance Time *via* OD_600_ Calculation and YFP Normalization

For kinetic optical density measurements in the presence of ampicillin, the transient tolerance time was calculated as the last local maximum in the log of the OD for each time series. To determine this, a moving window slope of 11 time points was used twice to calculate the first and second derivatives of the log OD. The last time point where the first derivative was closest to zero and where the second derivative was negative was taken as the tolerance time measured by OD_600_. Additionally, all fluorescence measurements after the measured tolerance time were normalized by the OD_600_ at the tolerance time to give an estimate of the FadD concentration which is unbiased by the reduction in OD due to cell death.

### Transcription Analysis by Reverse Transcription-qPCR

Cells were cultivated to steady state growth to an OD_600_ of 0.2 and 2 ml of cells were collected and stored in RNA/DNA Shield (Zymo Research, Irvine, CA, United States). Total RNA was extracted from cells using the Quick-RNA Miniprep Plus Kit (Zymo Research) following manufactures protocols. Contaminating genomic DNA was removed by DNase I treatment of RNA on collection column, following the Quick-RNA Miniprep Kit protocol. cDNA was synthesized using Revert Aid First strand cDNA Synthesis (Thermo Fischer Scientific) with random hexamer primers following the manufactures protocol. Negative control reactions without the use of reverse transcriptase were performed to evaluate the potential presence of contaminating genomic DNA. Two microliter of cDNA was amplified using Power SYBR green PCR Master Mix (Thermo Fischer Scientific) and gene specific primers (Supplementary Table S2). qPCR reactions for each biological replicate, gene, and growth condition were performed in triplicate. qPCR assays were performed on a QuantStudio3 (Thermo Fischer Scientific) following standard thermal cycling conditions recommended by the manufacturer. Expression levels of the condition invariant gene *gyrA* were used as a control for normalization between samples (Said-Salman et al., 2019). Fold changes of each gene of interest were calculated following the 2^−ΔΔCT^ method.

## Results

### Nutrient Shifts to Fatty Acid Stimulate Transient Ampicillin Tolerance

**Figure 1:**
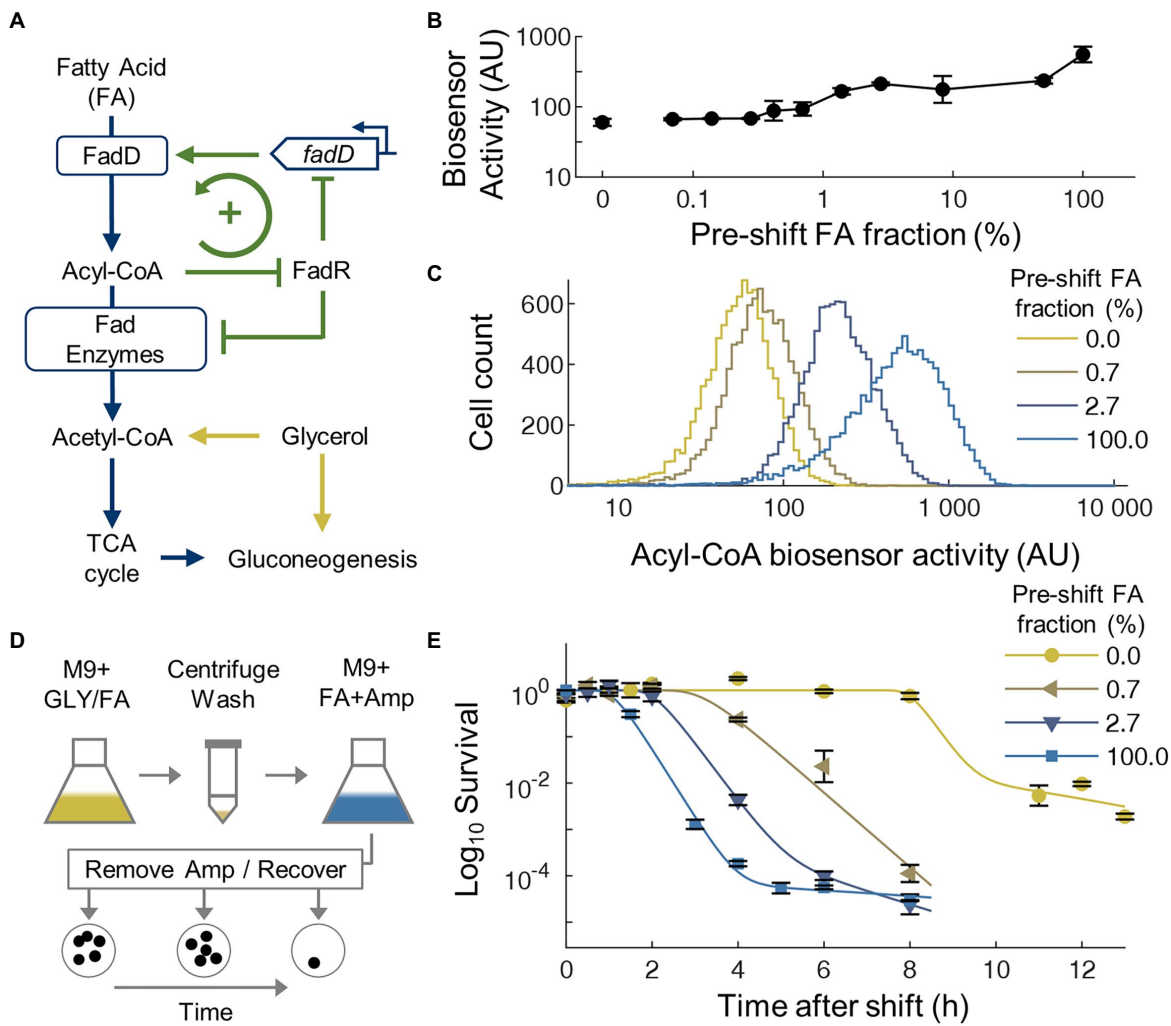
Transitions from glycerol to fatty acid (FA) generate transient ampicillin tolerance. **(A)** Simplified metabolic and regulatory network for FA and glycerol utilization. **(B)** Average acyl-CoA biosensor activity during co-utilization of FA and glycerol at different ratios. Across all conditions, the total concentration of carbon is constant at 72 mM. **(C)** Single-cell distribution of acyl-CoA biosensor activity during co-utilization of FA and glycerol at different ratios. A single representative distribution is shown per condition, *n* = 10,000 per distribution. All distributions are unimodal, with the mean shifting higher for higher fractions of FA. **(D)** Illustration of media switching and antibiotic killing experimental protocol. GLY, glycerol; Amp, ampicillin; and FA, fatty acid. **(E)** Time course survival curves of cells after nutrient shifts from mixtures of glycerol and FA to 100% FA with 100 μg/ml ampicillin. The glycerol/FA fraction in the pre-switch correspond with the conditions displayed for the distributions in **C**. Average of biological replicates, *n* = 3, error bars represent SEM. Curves are fitting of the Gompertz model to experimental data.

Similar to many other nutrients, FA uptake in *E. coli* is regulated by a positive feedback loop (Figure 1A). In this loop, the fatty acyl-CoA ligase FadD controls the uptake and activation of extracellular free FAs to acyl-CoAs, which are then catabolized by a series of FA degradation (Fad) enzymes to acetyl-CoA for use in central metabolism. Expression of *fadD* and the *fad* regulon is repressed by the transcriptional regulator FadR, whose DNA-binding activity is further inhibited by acyl-CoAs, forming a simple positive feedback loop. Modeling of this FA uptake loop shows that bistability in FA uptake can appear under some parameters and the parameters for *E. coli* may be near the bistable regime (Mannan and Bates, 2021). In the bistable regime, a sub-population of cells would have low FadD expression due to FadR repression, thus low FA uptake rate to activate FadR and to support rapid cell growth. Another sub-population would maintain a high FadD expression level, thus can keep high intracellular acyl-CoA level to both antagonizes FadR’s DNA-binding activity and to support cell growth. If bistability in FA uptake does occur, the rapid growing sub-population will be killed by ampicillin, whereas slow-growing cells may display tolerance to ampicillin which only targets growing cells.

To test the possible presence of bistability in FA uptake, we transformed *E. coli* cells with an engineered FadR-based acyl-CoA biosensor (Zhang et al., 2012) that reports intracellular acyl-CoA level (Xiao et al., 2016; Liu and Zhang, 2018) and indirectly reports expression of *fad* genes. To characterize the acyl-CoA biosensor activity under different conditions, the strain was grown to steady state in defined media containing mixtures of glycerol and oleate at different ratios. Flow cytometry showed that the mean activity of the acyl-CoA biosensor increased as the ratio of FA-to-glycerol increased (Figure 1B), consistent with the biosensor’s behavior in glucose/FA media (Liu and Zhang, 2018). At single-cell level, the biosensor activity showed unimodal distribution under all conditions (Figure 1C), without a distinctive sub-population above the detection limit of the method (~1% of the population). These results suggest that although positive FA uptake loop can support bimodality/bistability under some parameters (Mannan and Bates, 2021), the actual parameters for this strain of *E. coli* do not support a large bimodal population. Even so, a much smaller bimodal population may exist below the detection limit of flow cytometry.

Thus, to more sensitively determine whether two distinct sub-populations exist with different killing kinetics, we next conducted nutrient shifting experiments (Radzikowski et al., 2016) from different ratios of glycerol/FA mixtures to pure FA accompanied by ampicillin treatment (Figure 1D). When pure FA was used in the pre-culture as a negative control without nutrient switching (FA-to-FA), a typical bi-phasic killing kinetics was observed (Figure 1E): nearly immediate and rapid killing followed by a small population (0.008%) of antibiotic tolerant cells, close to previously reported levels of persistent cells in active cultures (Balaban et al., 2019). Although we observe a short period without killing (less than 1 h), which may be caused by starvation and cold stress from the washing procedures to provide temporary tolerance to ampicillin (Heinemann et al., 2020), this tolerance is not maintained for a large fraction of the population. Overall, this result proves that the switching procedure used in this study did not generate an elevated level of persisters. In contrast, when switching from glycerol to FA, the killing curve displayed much longer initial tolerant period, where nearly all cells survived ampicillin treatment during the first 8 h. This transient tolerance period is followed by a rapid killing where 98% of the transiently tolerant cells were killed. Finally, the rate of killing reduced with ~2% population having elevated antibiotic tolerance. When mixtures of glycerol and FA were used in the pre-culture, the killing curves also displayed an initial transient tolerant period followed by rapid killing. The length of this tolerance period increased as the amount of FA in the pre-culture decreased (Figure 1E). These results further confirm that the long transient tolerance to ampicillin was caused by specifically by nutrient shifting rather than the washing procedure used in this study and suggests a connection between the prior acyl-CoA activity with the tolerance period.

### Transient Tolerance Correlates With Lag Phase During Nutrient Shift

**Figure 2:**
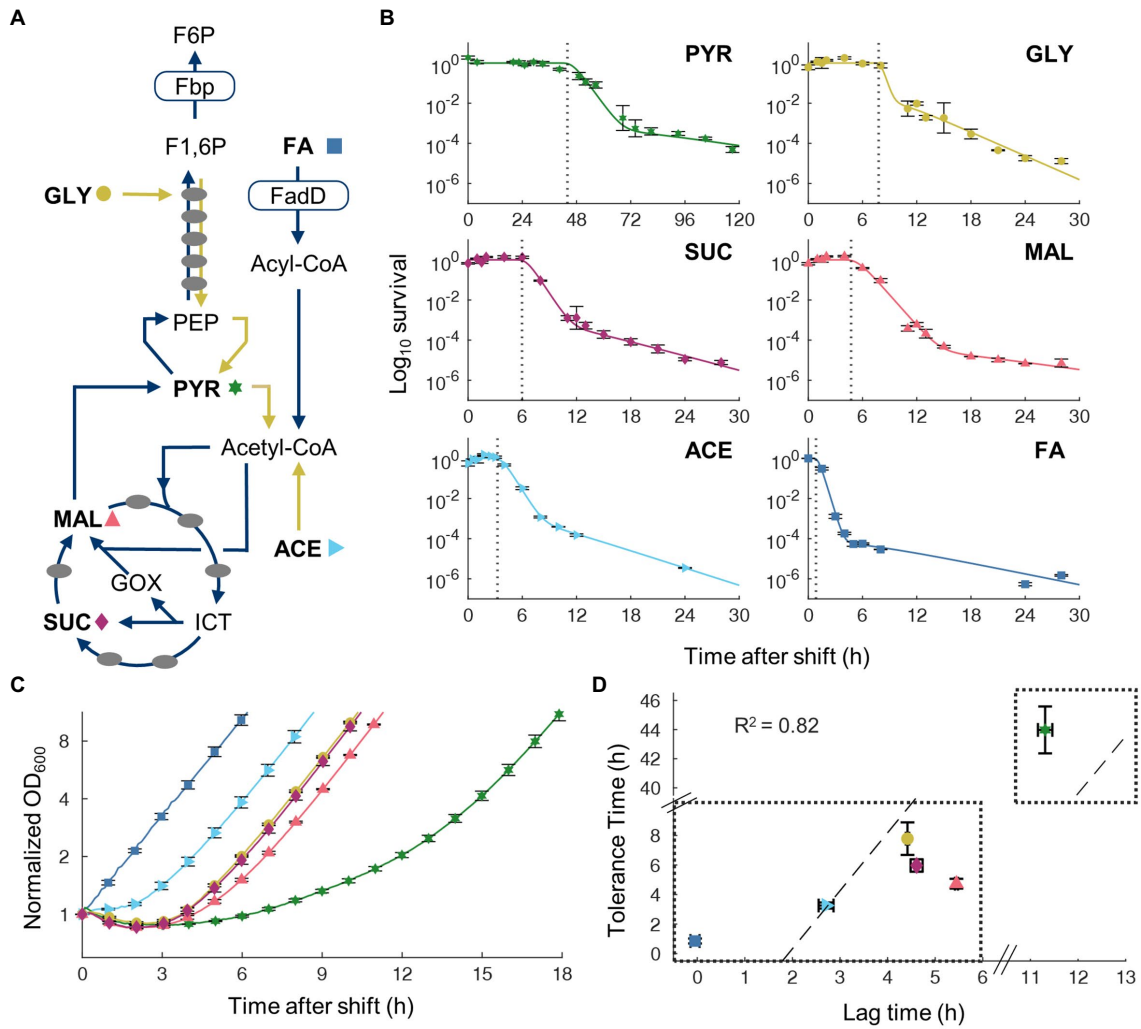
Tolerance time correlates with lag time on transitions from gluconeogenic carbon sources to oleate. **(A)** Simplified metabolic network for gluconeogenic carbon source utilization. Pre-shift carbon sources used are in bold with a corresponding symbol indicating that carbon source: (FA, square; acetate, right-pointing triangle; pyruvate, star; glycerol, circle; malate, up-pointing triangle; and succinate, kite). Dark arrows indicate pathways necessary for FA utilization. Names of key metabolite intermediates and enzymes are shown. **(B)** Time course survival curves of cells after shifts from gluconeogenic carbon sources to FA with 100 μg/ml ampicillin. Data points are averaged values from biological replicates, *n* = 3. Curves are fitting of a two-population Gompertz model to experimental data. Vertical dashed line indicates tolerance time determined by fitting the Gompetz model. **(C)** Time course OD_600_ after shift from gluconeogenic carbon to FA without ampicillin, normalized to time point *t* = 0. Average of biological replicates, *n* = 3, SEM error bars. **(D)** Correlation between measured tolerance time and lag time after switch to FA. Dashed line is least-squares linear fit to the data with equal weighting.

To examine whether the transient tolerant exists when switching from other gluconeogenic carbons, we performed additional switching experiment by replacing glycerol with four other carbon sources: acetate, pyruvate, malate, and succinate. These carbon sources represent different entry points into the central metabolism (Figure 2A), thus would allow us to compare how different metabolic state of cells in the pre-culture affect transient tolerance. All transitions displayed similar tri-phasic killing kinetics with an initial transient tolerance followed by rapid killing of susceptible cells and a slow killing of persistent cells. The period of transient tolerance varied significantly with different carbon sources (Figure 2B). While transitions from acetate had a relatively short transient tolerance time (3.3 ± 0.4 h, 95% CI), transitions from pyruvate displayed extremely long tolerance (44 ± 3 h, 95% CI). However, despite great variability in the timing, all conditions showed that majority of cells was eventually killed by ampicillin, indicating that the initial antibiotic tolerance is only temporary after gluconeogenic-to-FA nutrient shifts.

Shifts from glycolytic to gluconeogenic carbon sources have been shown to produce lag phase during which cell halts growth transiently (Basan et al., 2020). Since ampicillin is only effective on growing cells, cessation of growth after a nutrient shift offers a simple mechanism for transient tolerance. To test this, we measured growth kinetics of cultures switching from each gluconeogenic carbon source to FA without antibiotic (Figure 2C). The lag time of each shift was found to correlate well (*R*^2^ = 0.82) with the transient tolerance time (Figure 2D). These results suggest that non-growing cells in the lag phase after a nutrient shift cause transient tolerance to ampicillin.

### Time for FadD Accumulation Correlates With Transient Tolerance

**Figure 3:**
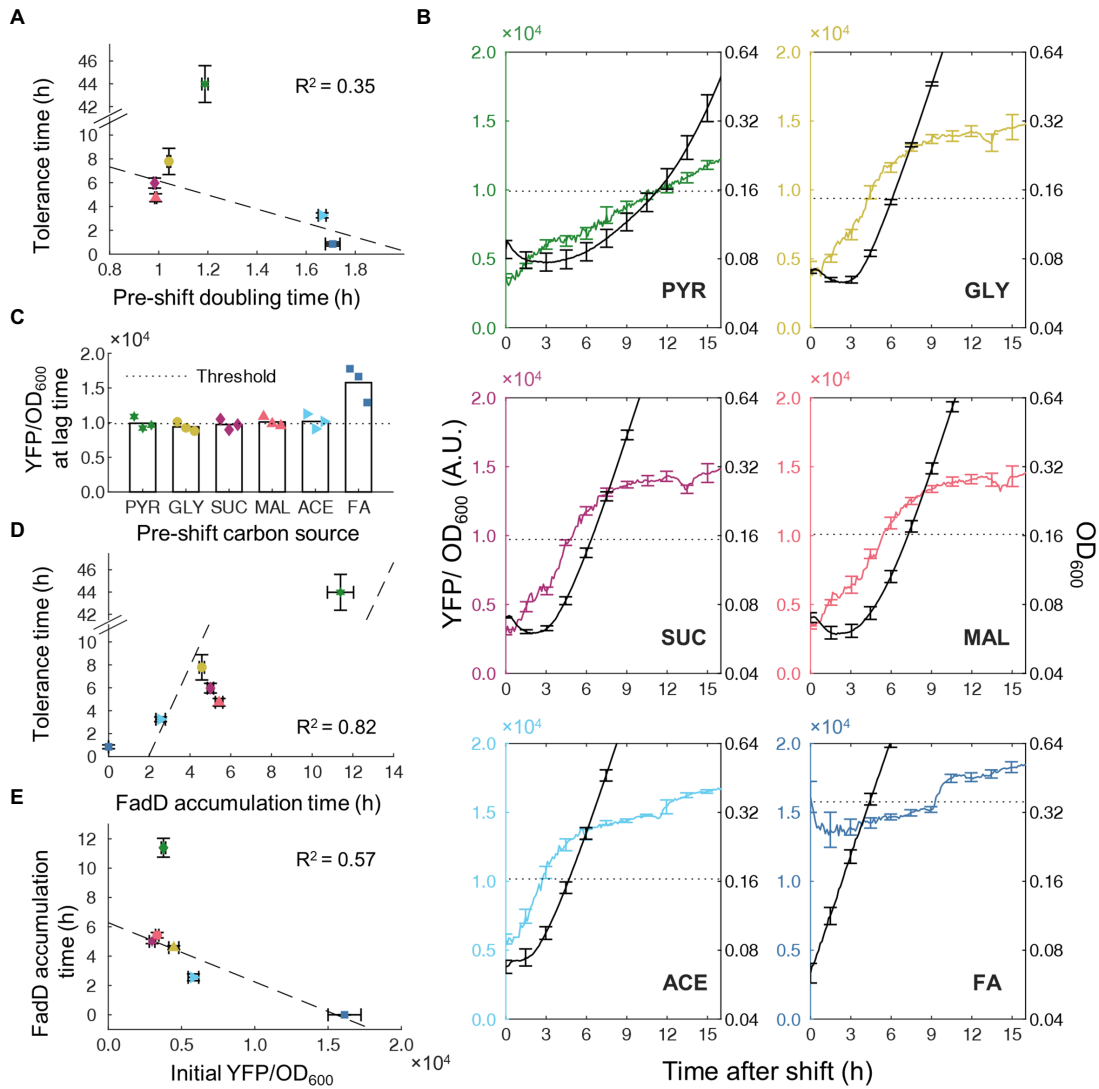
Timing of FadD accumulation associated with transient tolerance time. **(A)** Correlation between measured tolerance time and growth rate in pre-switch carbon source. Dashed line is least-squares linear fit to data with bisquare weighting. **(B)** Time course of YFP/OD_600_ from FadD-YFP fusion (colored line, left axis) and OD_600_ (black line, right axis) after shifts from a gluconeogenic carbon source to FA, *n* = 3, Error bars represent SEM. The dotted line indicates the YFP/OD_600_ level when cells resume steady state growth. **(C)** Average YFP/OD_600_ from FadD-YFP fusion at the end of the lag phase, *n* = 3. Values from individual replicates shown as points. The dashed line (namely the FadD threshold) shows the average FadD concentration at the end of lag phase across all pre-shift conditions except FA. **(D)** Correlation between tolerance time and FadD accumulation time (time for FadD to reach the threshold level). Tolerance time was obtained from data in Figure 2B. Dashed line is a linear fit. **(E)** Correlation between YFP/OD_600_ value at the steady state of pre-culture and FadD accumulation time. Dashed line is a least-squares linear fit with bisquare weighting.

Since the lag time and transient tolerance period after the nutrient shift are well correlated, we sought to understand the molecular mechanisms which govern both phenomena. Growth rate in pre-culture conditions was shown to predict lag time for glycolytic-to-gluconeogenic switches (Basan et al., 2020) and therefore may also predict tolerance time on nutrient shifts from gluconeogenic carbons to FA. We measured pre-culture growth rates and found a weak negative correlation (*R*^2^ = 0.35) between pre-culture growth rate and tolerance time (Figure 3A). Although the direction of correlation is consistent with previous studies (Basan et al., 2020), nutrient shifts from pyruvate to FA present a clear outlier, suggesting the presence of other mechanisms that control tolerance time more directly.

Previous studies illustrated that β-oxidation enzymes are essential for growth when FA is the sole carbon source (DiRusso et al., 1999), suggesting a possible role for β-oxidation enzymes in tolerance time during nutrient shifts to FA. Because FadD is a key component of the positive feedback loop controlling transport and activation of FAs for β-oxidation, we genetically fused a YFP to FadD to monitor activity of FA degradation during the nutrient shift. The FadD-YFP strain was grown in different gluconeogenic carbon sources and switched to FA in the absence of ampicillin. Cell growth and YFP fluorescence were simultaneously monitored after the nutrient shift. After shifting from different gluconeogenic carbon sources, the initial FadD levels were different and all lower than that without carbon shift (i.e., from FA to FA, Figure 3B), suggesting that the β-oxidation enzymes were not highly expressed in gluconeogenic carbons. The FadD concentration gradually increased over time during the lag phase. When cells resume steady state growth, FadD concentration from all switches reached the same threshold level (Figure 3C). Without carbon shift (from FA to FA), the FadD level remained above this threshold level, and the cells continued to grow without a lag phase. We measured the time needed to reach this threshold FadD level and found that it has a good correlation with the tolerance time (*R*^2^ = 0.82; Figure 3D). Since the timing of accumulating FadD correlates well with transient tolerance, we wondered whether this timing of each phenomenon is simply determined by the amount of FadD initially present immediately after the nutrient shift. Although FadD accumulation time appears to decrease with increasing initial FadD concentrations (*R*^2^ = 0.57; Figure 3E), transitions from pyruvate again provide a strong outlier with cells taking much longer to accumulate FadD to a threshold.

To verify whether these FadD dynamics are representative of the nutrient shifts in the presence of antibiotics, we performed similar experiments using the FadD-YFP fusion strain in the presence of ampicillin. Across all switching conditions, OD_600_ initially decreased slightly followed by a leveling off period (Supplementary Figure S1A). Because this slight decrease is also observed in the absence of antibiotic, this decrease more likely represents reductive division of cells, which has been previously reported for cells under starvation conditions (Nyström, 2004), rather than killing by antibiotics. After this relatively level period (transient tolerance), the OD decreases substantially, corresponding to rapid killing of susceptible cells. In all switching conditions, the FadD level continued to increase during the transient tolerance period, indicating active transcription and translation. The time needed to reach a threshold FadD level still correlated well with the tolerance time (*R*^2^ = 0.79, Supplementary Figure S1B). Additionally, the timing of FadD accumulation in the absence and presence of ampicillin correlated extremely well (*R*^2^ = 0.997; Supplementary Figure S1C), indicating that the FadD dynamics during the nutrient shift are nearly the same prior to reaching the FadD threshold, regardless of the presence of ampicillin. Overall, these results suggest that FA metabolic activity was low during lag phase, resulting in slow cell growth and transient tolerance to ampicillin. A key level of FadD is needed to resume cell growth and active metabolism in FA medium.

Finally, we evaluated the single-cell behavior of FA metabolism using the acyl-CoA biosensor during nutrient transitions from pyruvate to FA, which has the longest transient tolerance time. We characterized the acyl-CoA biosensor activity under mixtures of pyruvate and FA and found that the population has a monomodal distribution of acyl-CoA biosensor activity under all conditions (Supplementary Figure S2A), consistent with the biosensor activity on glycerol/FA mixtures (Figure 1C). We then conducted nutrient switches from pyruvate to FA with ampicillin and measured the acyl-CoA biosensor activity at time points during the transient tolerance phase. Again, we observed monomodal populations at all-time points, with the biosensor activity of the whole population increasing sharply by 4 h after the shift, and then more gradually after 21 h (Supplementary Figure S2B). Thus, these results suggest that there is a single, large majority population before the shift and this whole population gradually transitions toward active FA metabolism after the shift, leading to growth and sudden killing by ampicillin for a large population at the end of the transient tolerance.

### Multiple Metabolic Regulations Control the Transient Tolerance Time

**Figure 4:**
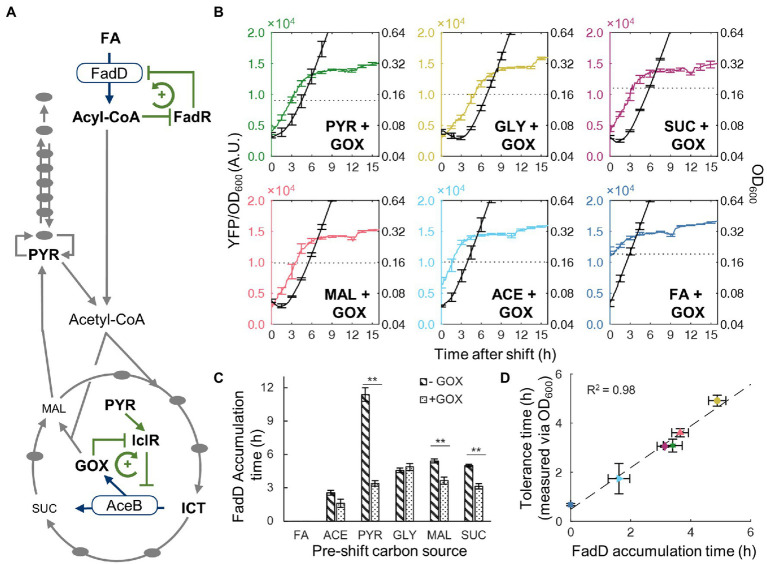
Activity of glyoxylate shunt affects tolerance time. **(A)** Simplified metabolic network with key positive feedback regulatory interactions for FA utilization. Metabolic enzymes (FadD/AceB) convert a precursor into a regulatory metabolite intermediate (acyl-CoA/glyoxylate). The regulatory intermediate represses the activity of a transcription factor (FadR/IclR) which alleviates the repression of the metabolic enzyme. IclR activity is also activated by pyruvate. **(B)** Time course of YFP/OD_600_ from FadD-YFP fusion (colored line, left axis) and OD_600_ (black line, right axis) after shifts from a glyoxylate supplemented gluconeogenic carbon source to FA (not supplemented with glyoxylate). Error bars represent SEM with *n* = 3 for FA, ACE, MAL, and SUC, *n* = 4 for PYR, and *n* = 5 for GLY. The dotted line indicates the YFP/OD_600_ level when cells resume steady state growth. **(C)** FadD accumulation time for nutrient shifts from carbon source without glyoxylate (stripes) or with glyoxylate (dotted). Stars indicate significant change in accumulation time, two-tailed *t*-test (**, *p* < 0.01). **(D)** Correlation between tolerance time and FadD accumulation time for shifts from carbon with glyoxylate to FA. Tolerance timed determined by time course optical density measurements (Supplementary Figure S4.) Dashed line is a least-squares linear fit.

Given that the time for FadD to reach its threshold concentration correlates well with transient tolerance, we wondered what controls FadD accumulation. Particularly, shifts from pyruvate to FA produced the longest transient tolerance times from the above experiment. We searched the literature and did not find direct regulatory effects of pyruvate on *fadD* expression in *E. coli*. However, pyruvate activates the IclR transcription factor (Lorca et al., 2007) which represses expression the glyoxylate bypass operon *aceBAK* (Figure 4A). The glyoxylate bypass is necessary for FA utilization because β-oxidation produces only two-carbon metabolite precursors in the form of acetyl-CoA (Dolan and Welch, 2018). To examine whether pyruvate impacts the glyoxylate bypass or β-oxidation gene expression, we evaluated the expression level of the *aceB* and *fadD* genes *via* RT-qPCR for cells growing in glycerol, pyruvate, and FA (Supplementary Figure S3). In pyruvate, *aceB* expression is 1.7-fold lower compared to that in glycerol; however, *fadD* expression is not significantly different between glycerol and pyruvate conditions, indicating that the glyoxylate bypass rather than the FA transport genes is more blocked in pyruvate. During growth on FA, both *aceB* and *fadD* expression are significantly increased (13.7-fold and 5.0-fold increase, respectively), which indicates that the expression of both *aceB* and *fadD* genes needs to be increased for cells to grow on FA, in agreement with previous literature (Clark and Cronan, 2005).

In addition to pyruvate regulation, IclR activity is antagonized by glyoxylate (Lorca et al., 2007), an intermediate in the glyoxylate bypass (Figure 4A), thus forming a positive feedback loop which also governs FA utilization. Therefore, when cells are growing in pyruvate, accumulation of glyoxylate is slow, and the glyoxylate bypass enzymes remain at low levels. We hypothesize that switching from pyruvate to FA requires a gradual increase in the metabolic flux through the glyoxylate bypass to produce central metabolites (e.g., amino acids) for synthesizing β-oxidation enzymes. To test this hypothesis, we added glyoxylate to the pre-shift medium to activate the glyoxylate bypass enzymes, thus alleviating the bottleneck in the glyoxylate bypass enzymes. New nutrient shift experiments with glyoxylate displayed decreased lag phase as well as FadD accumulation times for pyruvate, succinate, and malate (Figures 4B,C). Among them, pyruvate exhibited the most drastically change, its FadD accumulation time decreased from 11.4 ± 1.3 h to 3.4 ± 0.5 h (95% CI; Figure 4C). Additionally, we performed similar growth kinetic experiments during nutrient shift to FA with ampicillin and observed similar reductions in the transient tolerance time measured by OD_600_ (Supplementary Figure S4). The transient tolerant time shifting from pyruvate with glyoxylate supplement to FA decreased from >16 h without glyoxylate to 3.1 ± 0.5 h (95% CI). With the presence of glyoxylate in the pre-shift medium, the highest correlation between the transient tolerance period and FadD accumulation time was obtained (*R*^2^ = 0.98; Figure 4D), demonstrating that both FadD accumulation and transient tolerance are accelerated by the removal of the glyoxylate bottleneck.

## Discussion

In this study, we sought to elucidate the role of nutrient shifts and positive metabolic feedback architecture in producing antibiotic tolerant cells. In doing so, we discovered that nutrient shifts from gluconeogenic carbon source to FA produce tri-phasic antibiotic killing kinetics which depend on the initial metabolic state of the cells. These killing kinetics are defined by an initial period of nearly universal antibiotic survival, followed by a sudden increase in antibiotic killing for over 98% of the population, followed by a slower, persister-killing rate. Our results outline a clear mechanism for this behavior. First, before switching to FA, cells are in single population of low Fad activity. After shifting to FA, the bulk of the population is in a metabolically mal-adapted state which causes the cells to cease growth, producing a lag phase. In this metabolically reduced stated, ampicillin, which targets actively dividing cells, is rendered ineffective. This tolerance continues until the cells adapt their metabolism by increasing the concentration of key metabolic enzymes such as the FA degradation and glyoxylate bypass pathways to restore metabolism and growth. Further, the timing of the recovery depends on its initial metabolic state and on specific regulatory mechanisms for enzyme induction.

The connection between lag phase and antibiotic tolerance has been demonstrated previously (Fridman et al., 2014) when shifting from stationary phase media to rich media. In the so-called “tolerance by lag” (TBL) phenotype, mutant strains of *E. coli* have a population of cells with a wide distribution of lag times when shifting back to rich media, which allows the population to be more tolerant to ampicillin killing. While TBL and transient tolerance can both be attributed to the growth-arrest of cells in a lag phase, there are several important distinctions between these phenomena. First, the shapes of the killing curves are different. In transient tolerance, the killing curve is initially flat followed by a sharp decline which suggests that transient tolerant populations shift from high tolerance to low tolerance over time. In contrast, the TBL killing curves gradually decrease at a constant slope without distinct phases which suggests that tolerance from TBL is more constant in time. Second, TBL is caused by mutations in genes which are selected for by the length of antibiotic treatments. In contrast, transient tolerance is caused by a major disruption of the metabolic network due to nutrient shift which forces cells to halt growth. Finally, although transient tolerance is correlated with lag, we show that transient tolerance is more fundamentally affected by the time is takes to readjust the metabolic network by accumulating pathway enzymes. Because nutrient shifts can often leave cells in a metabolically mal-adapted state, transient tolerance is likely to be a general tolerance mechanism to ampicillin for cell populations.

Since the FA utilization and β-lactam tolerance mechanisms in *E. coli* are well known, the connection between adaptation of the FA pathways and transient tolerance may seem apparent at first. However, the tri-phasic shape of the antibiotic killing curves shed important insights and raise new questions about how *E. coli* responds to nutrient shifts in general. In particular, it has previously been demonstrated that on glucose-to-fumarate transitions cells adopt a responsive diversification strategy where only a small minority of cells are capable of adaptation and the vast majority becoming persisters (Kotte et al., 2014; Radzikowski et al., 2016). From these experiments, it has been suggested then that prolonged cold shock and flux limitation are triggers of persistence (Heinemann et al., 2020). Our results show that cold shock and flux limitation on nutrient shifts do not necessarily trigger persistence but can instead induce only a temporary tolerance. The correlation of transient tolerance with adaptation to FA utilization and the sudden killing of at least 98% of the population suggests that a majority of cells adapt to utilizing FA after a gluconeogenic-to-FA transitions with very few cells entering a long-lived persister state. Therefore, our results demonstrate that despite both being regulated by positive feedback, glycolytic-to-gluconeogenic and gluconeogenic-to-FA transitions follow fundamentally different adaptation strategies. These contrasting results highlight the need for a deeper understanding of how the underlying molecular mechanisms contribute to the choice of adaptation strategies in response to nutrient transitions.

More generally, our results elucidate the role of metabolic positive feedback loops in nutrient shift and β-lactam tolerance. For example, the positive feedback loop in the glyoxylate bypass causes cells to maintain transient tolerance for up to 44 h when switching from pyruvate to FA. Adding glyoxylate to the pre-shift medium accelerated both transient tolerance and FadD accumulation, indicating that a bottleneck in the glyoxylate bypass has a global effect on the metabolic network, beyond its own regulatory loop. Previous studies of antibiotic persistence have demonstrated the possibility of potentiating antibiotic killing by introducing key metabolites (Allison et al., 2011). Our results demonstrate that glyoxylate can act as a key regulatory metabolite to prime the metabolic network to adapt to certain nutrient shifts, thus reducing transient tolerance to β-lactams. Because of the presence of these specific metabolic regulations, cells maintain a memory of their pre-shift nutrient conditions, causing large differences in tolerance time despite having identical post-shift nutrient environments. Additionally, although we mainly focus on understanding the transient tolerance period, we note that shifts from different carbon sources also have different rates of antibiotic killing and different persister fractions (Figure 2B; Supplementary Table S1). These observations suggest a more profound impact of metabolism on persistence, which are worth further studies. Altogether, our findings show how the pre-shift metabolic conditions can have long lasting effects on the metabolism and antibiotic tolerance of cells after a nutrient shift.

## Data Availability Statement

The original contributions presented in the study are included in the article/Supplementary Material; further inquiries can be directed to the corresponding author.

## Author Contributions

CH and FZ designed the experiments, analyzed the data, and wrote the paper with feedback from RZ. CH and RZ performed the experiments. All authors contributed to the article and approved the submitted version.

## Funding

This work is supported by the National Institute of General Medical Sciences of the National Institutes of Health under Award Number R35GM133797.

## Conflict of Interest

The authors declare that the research was conducted in the absence of any commercial or financial relationships that could be construed as a potential conflict of interest.

## Publisher’s Note

All claims expressed in this article are solely those of the authors and do not necessarily represent those of their affiliated organizations, or those of the publisher, the editors and the reviewers. Any product that may be evaluated in this article, or claim that may be made by its manufacturer, is not guaranteed or endorsed by the publisher.

## Abbreviations

ACE: Acetate
Amp: Ampicillin
FA: Fatty acid
F1,6P: Fructose-1,6-bisphosphate
F6P: Fructose 6-phosphate
GLY: Glycerol
GOX: Glyoxylyate
ICT: Isocitrate
MAL: Malate
PEP: Phosphoenolpyruvate
PYR: Pyruvate
SUC: Succinate
TBL: Tolerance by lag
