# Supplementary material for: Transient Antibiotic Tolerance Triggered by Nutrient Shifts From Gluconeogenic Carbon Sources to Fatty Acid
Source: Front Microbiol. 2022 Mar 11;13:854272. doi: 10.3389/fmicb.2022.854272 (PMC8963472; doi:10.3389/fmicb.2022.854272)
Supplement: Supplementary file 1 [file Data_Sheet_1.DOCX]

**Supplemental Information**

**Table of Contents**

**Figure S1. FadD accumulation dynamics in the presence of ampicillin after nutrient shift**

**Figure S2. Single-cell distribution of acyl-CoA biosensor activity during pyruvate to FA shifts**

**Figure S3. Pre-shift relative transcription levels for *aceB* and *fadD* genes.**

**Figure S4. FadD accumulation dynamics in the presence of ampicillin after nutrient shift with pre-shift glyoxylate**

**Table S1. Fitted parameters for two-population Gompertz model**

**Table S2. Strains, plasmids, and primers used in this study**

**Table S3. DNA sequences of plasmids and strains constructed in this study**

**
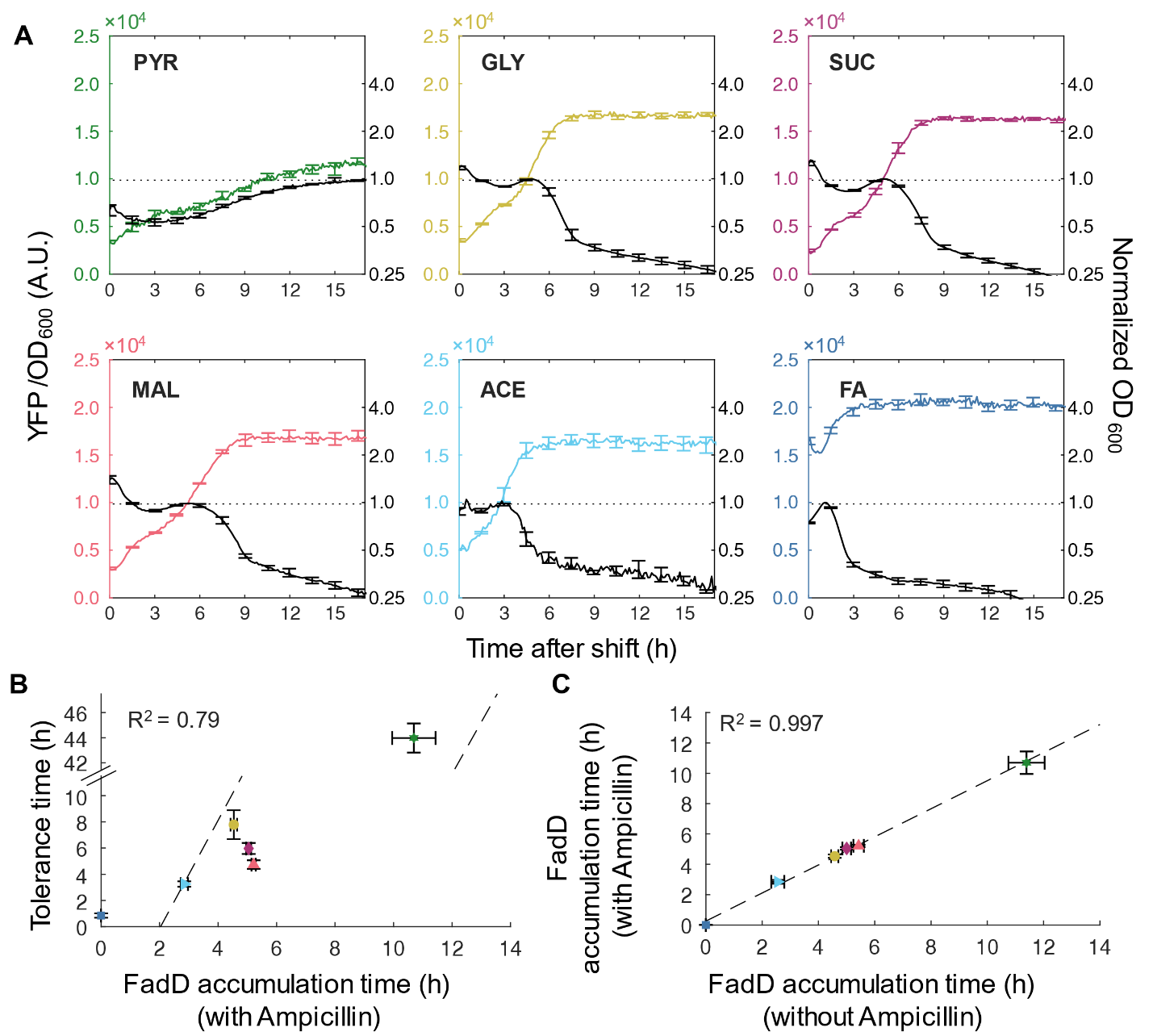
**

**Figure S1. FadD accumulation dynamics in the presence of ampicillin after nutrient shift.**

**(A)** Time course of YFP/OD_600_ from FadD-YFP fusion (colored line, left axis) and OD_600_ (black line, right axis) after switches from carbon sources to FA with ampicillin, n = 3, Error Bars represent SEM. The dotted line indicates the FadD threshold as determined in Fig 3B, OD_600_ data normalized to the OD_600_ at the tolerance time determined by OD_600_.

**(B)** Correlation between tolerance time and time for FadD to accumulate to the FadD threshold (accumulation time) in the presence of ampicillin. Tolerance time data calculated from data in Fig 2B. Dashed line is a linear fit.

**(C)** Correlation between FadD accumulation time in the presence and absence of ampicillin. Dashed line is linear fit to data.


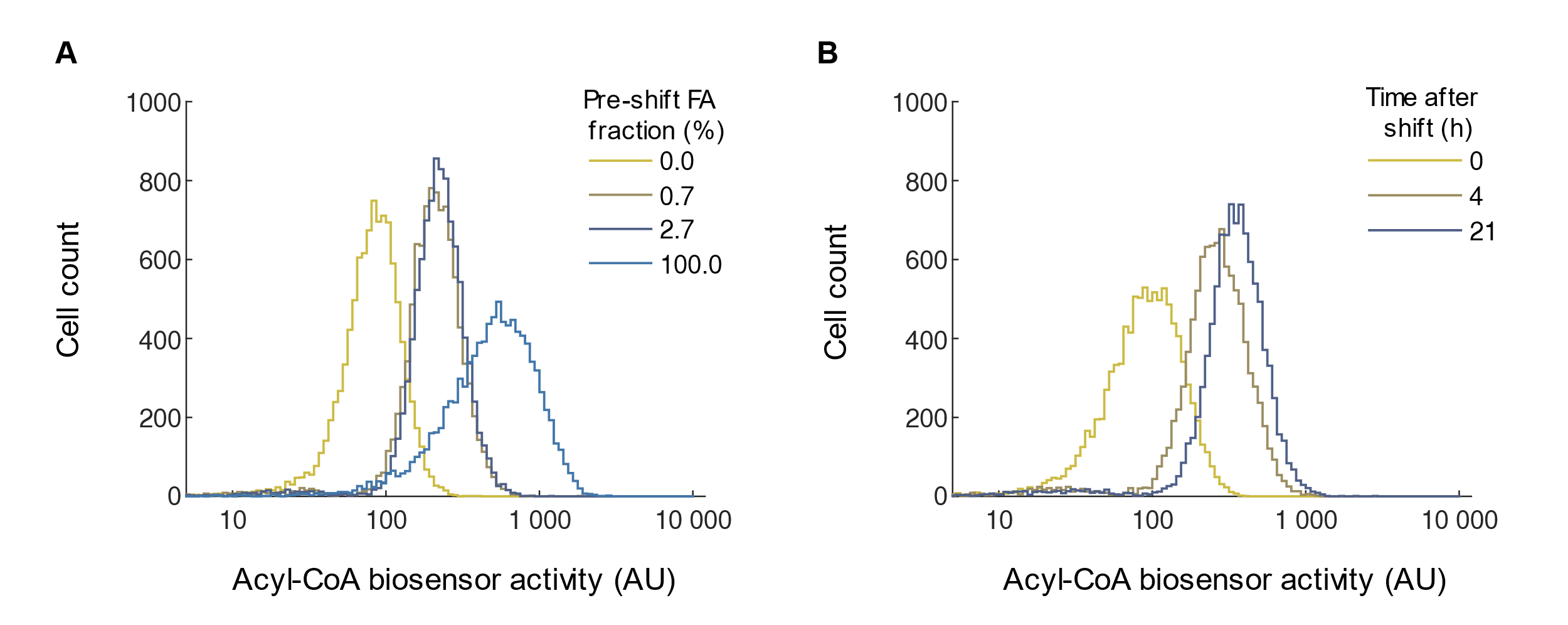


**Figure S2. Single-cell distribution of acyl-CoA biosensor activity during pyruvate to FA shifts**

**(A)** Single-cell distribution of acyl-CoA biosensor activity during co-utilization of FA and pyruvate at different ratios. A single representative distribution is shown per condition, n = 10,000 per distribution. All distributions are unimodal, with the mean shifting higher for higher fractions of FA.

**(B)** Time course single-cell distribution of acyl-CoA biosensor activity after shift from pyruvate to FA with ampicillin. A single representative distribution is shown per time point, n = 10,000. All distributions are monomodal, with mean biosensor activity increasing over time after shift to FA. The acyl-CoA biosensor kinetics after the shift are consistent with the FadD kinetics measured on plate reader (Fig. S1, pyruvate).


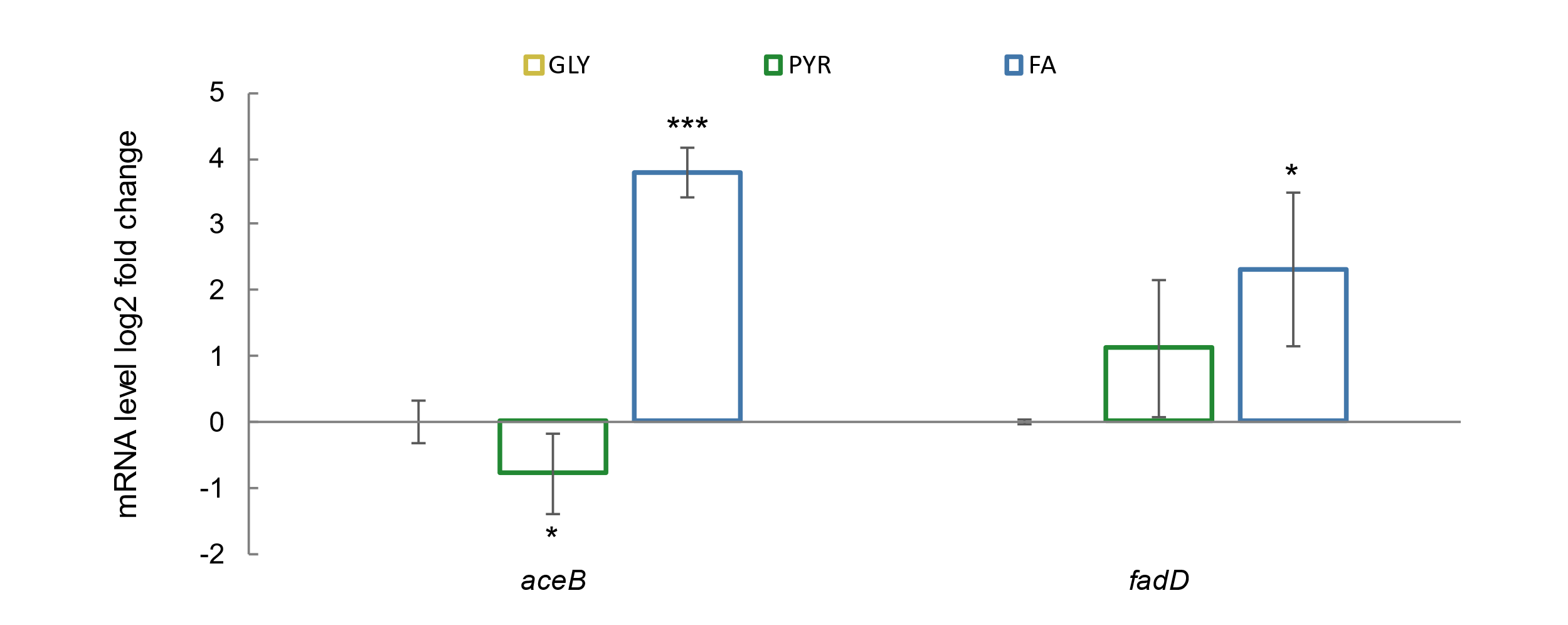


**Figure S3. Pre-shift relative transcription levels for *aceB* and *fadD* genes.** mRNA levels of genes measured from steady state cultures growing in glycerol (left, yellow), pyruvate (middle, green), or FA, (right, blue). Transcription levels are relative to pre-shift growth in glycerol. n = 3 biological replicates, Error bars represent standard deviation. Stars indicate significant change in transcription compared to glycerol, two-tailed t-test (*, p < 0.05, *** p <0.001)

**
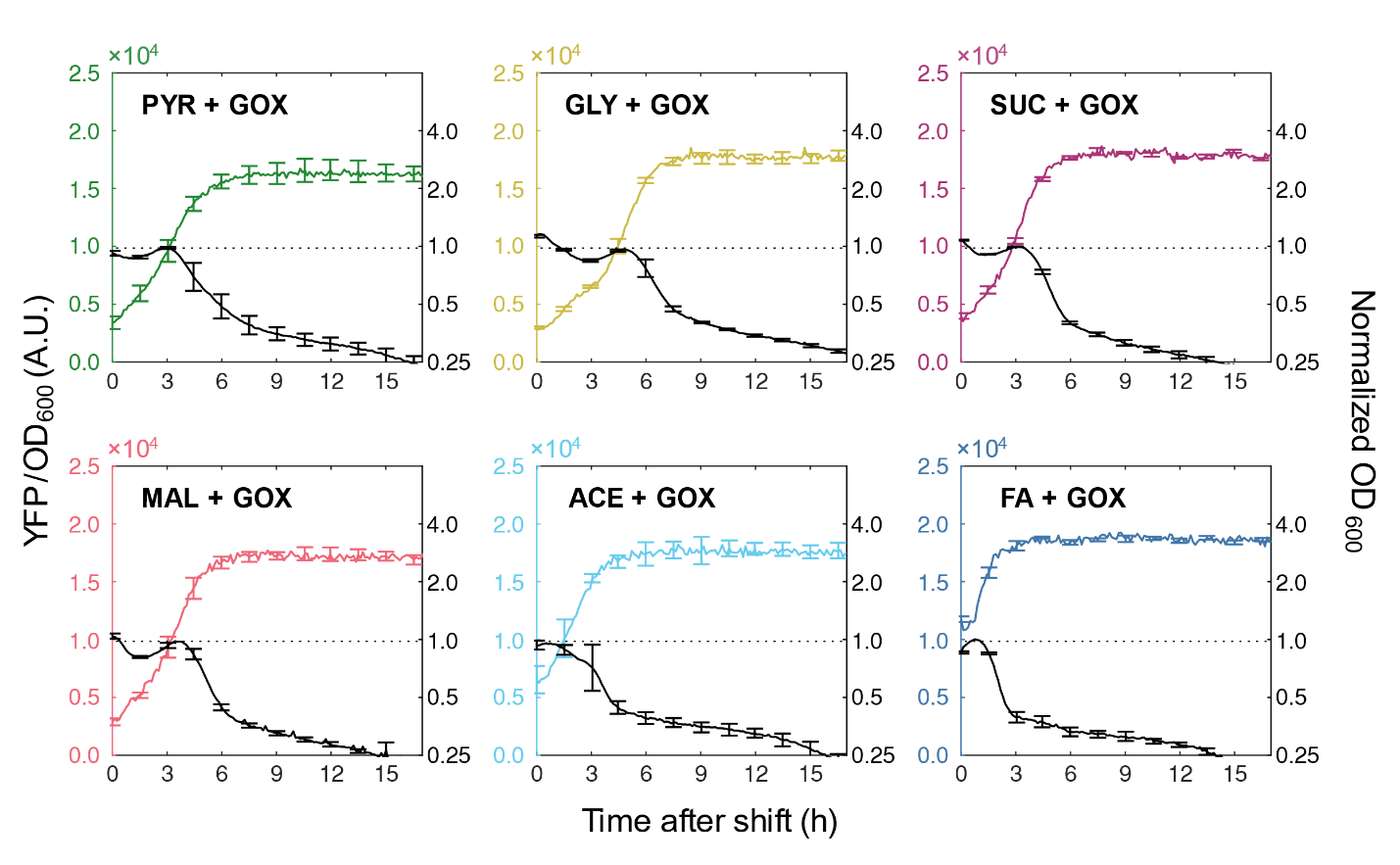
**

**Figure S4. FadD accumulation dynamics in the presence of ampicillin after nutrient shift with pre-shift glyoxylate.** Time course of YFP/OD_600_ from FadD-YFP fusion (colored line, left axis) and OD_600_ (black line, right axis) after shifts from a glyoxylate supplemented gluconeogenic carbon source to FA with ampicillin (not supplemented with glyoxylate), n = 3, Error Bars represent SEM. The dotted line indicates the FadD threshold as determined in Fig 3B, OD_600_ data is normalized to the OD_600_ at the tolerance time determined by OD_600_ (Note: Normalized OD_600_ serves only as approximate measure of cell survival, since OD accurately determine the fraction of live and dead cells).

**Table S1.** **Fitted parameters for two-population Gompertz model**

Model parameters are given along with their 95% confidence intervals, N.D.: Not Determined

| **Pre-Shift Media** | **Tolerance Time (τ)**  **(hours)** | **Transient Tolerant Killing Rate (μ_TT_)**  **(hour^-1^)** | **Persister Killing Rate (μ_TT_)**  **(hour^-1^)** | **Log10 Persister Fraction (log10(p))** |
| --- | --- | --- | --- | --- |
| **4mM FA** | 0.9 ± 0.3 | 1.25 ± 0.22 | 0.076 ± 0.011 | -4.1 ± 0.2 |
| **24 mM Acetate** | 3.3 ± 0.4 | 0.62 ± 0.09 | 0.122 ± 0.014 | -2.9 ± 0.2 |
| **18 mM Malate** | 4.7 ± 0.6 | 0.43 ± 0.05 | 0.053 ± 0.023 | -4.3 ± 0.4 |
| **18 mM Succinate** | 6.0 ± 0.8 | 0.59 ± 0.14 | 0.101 ± 0.020 | -3.1 ± 0.3 |
| **24 mM Glycerol** | 7.8 ± 2.2 | 1.22 ± 11.91 | 0.167 ± 0.025 | -1.9 ± 0.3 |
| **24 mM Pyruvate** | 44.0 ± 3.2 | 0.13 ± 0.04 | 0.015 ± 0.008 | -3.2 ± 0.4 |
| **23.8 mM Glycerol, 0.03 mM FA** | 2.7 ± 0.4 | 0.68 ± 0.07 | N.D. | N.D. |
| **23.3 mM Glycerol,**  **0.11 mM FA** | 1.7 ± 0.4 | 1.06 ± 0.23 | 0.249 ± 0.156 | -3.2 ± 0.9 |

**Table S2. Strains, plasmids, and primers used in this study**

| **Strain** | **Genotype** | **Source or Reference** |
| --- | --- | --- |
| NCM3722 (WT) | F^+^ | CGSC#:12355 |
| Acyl-CoA-Biosensor Strain | NCM3722 pSARk-yemGFP | This work |
| FadD-YFP Strain | NCM3722 φ(*fadD-YFP*) | This work |
|  | | |
| **Plasmids** | **Genotype** | **Source** |
| pSARk-yemGFP | SC101 ori, KAN^R^, P_AR_-*yemgfp* | This work |
| pTargetF-FadD-YFP | colE1 ori, SPEC^R^, | This work |
|  | | |
| **Cloning primers** | **Sequence** |  |
| yemGFP_F | TTCAAAAGATCTTTTAAGAA  GGAGATATACATATGTCTAA  AGGTGAAGAATTATTCAC | This work |
| yemGFP_R | GAGTTTGGATCCTTATTTGTA  CAATTCATCCATACC | This work |
| pTargetF_FadDN20_F | ggcattggtctcggacttaac  gctcgttttagagctagaaat  agcaagttaaaataa | This work |
| pTargetF_FadDN20_R | TTCCACGGTCTCCccagatct  aagcttctgcagg | This work |
| pTargetF_FadD_  upstream_F | TTCCACGGTCTCCctggtttta  acgtctatcccaac | This work |
| pTargetF_FadD_  upstream_16L_part1_R | ccgccgccgttagagggagtg ctaccaccggctttattgtcca  ctttgcc | This work |
| pTargetF_FadD_  upstream_16L_part2 | ACAGCTGGTCTCCctaccaccg  ctgccgctgccgtcgtcgccgc  cgccgttagaggg | This work |
| pTargetF_YFP_F | ACAGCTGGTCTCCgtagcaaag  gtgaagaactgttcac | This work |
| pTargetF_YFP_R | TTCCACGGTCTCCcattatttat  acagttcgtccataccc | This work |
| pTargetF_FadD_  downstream_F | TTCCACGGTCTCGaatgagcgt  taagtcagtcg | This work |
| pTargetF_FadD_  downstream_R | TTCCACGGTCTCGagaacccca  gctgcgggtaa | This work |
| pTargetF_  Backbone_F | TTCCACGGTCTCGttctcgagtt  catgtgcagc | This work |
| pTargetF_  Backbone_R | GGCATTGGTCTCGAGTCAGTCG  TCAactagtattatacctaggactgagc | This work |
|  | | |
| **qPCR primers** | **Sequence** | **Source of Reference** |
| gyrA_F | Gtcgtggcgggaaaggtaaa |  |
| gyrA_R | CGGCTGGAGAAGCACAGAA |  |
| aceB_F | ACCTTGTGATGGTGAACGCA |  |
| aceB_R | TCCACTGCCAGATCGAGGTA |  |
| fadD_F | CCGATCAACCTGCGTTTGTG |  |
| fadD_R | TTCTTCAGCCCCAACCCTTG |  |

**Table S3. DNA sequences of plasmids and strains constructed in this study**

| **DNA** | **Sequence** | **Feature Notes** |
| --- | --- | --- |
| pSARk-yemGFP | aaaatttatcaaaaagagtgttgactatctggtacgaccagatgatacttagattcatctggtacgaccagataccgaattcaaaagatcttttaagaaggagatatacatatgtctaaaggtgaagaattattcactggtgttgtcccaattttggttgaattagatggtgatgttaatggtcacaaattttctgtctccggtgaaggtgaaggtgatgctacttacggtaaattgaccttaaaatttatttgtactactggtaaattgccagttccatggccaaccttagtcactactttaacttatggtgttcaatgtttttctagatacccagatcatatgaaacaacatgactttttcaagtctgccatgccagaaggttatgttcaagaaagaactatttttttcaaagatgacggtaactacaagaccagagctgaagtcaagtttgaaggtgataccttagttaatagaatcgaattaaaaggtattgattttaaagaagatggtaacattttaggtcacaaattggaatacaactataactctcacaatgtttacatcatggctgacaaacaaaagaatggtatcaaagttaacttcaaaattagacacaacattgaagatggttctgttcaattagctgaccattatcaacaaaatactccaattggtgatggtccagtcttgttaccagacaaccattacttatccactcaatctaaattatccaaagatccaaacgaaaagagagaccacatggtcttgttagaatttgttactgctgctggtattacccatggtatggatgaattgtacaaataaggatccaaactcgagtaaggatctccaggcatcaaataaaacgaaaggctcagtcgaaagactgggcctttcgttttatctgttgtttgtcggtgaacgctctctactagagtcacactggctcaccttcgggtgggcctttctgcgtttatacctagggtacgggttttgctgcccgcaaacgggctgttctggtgttgctagtttgttatcagaatcgcagatccggcttcagccggtttgccggctgaaagcgctatttcttccagaattgccatgattttttccccacgggaggcgtcactggctcccgtgttgtcggcagctttgattcgataagcagcatcgcctgtttcaggctgtctatgtgtgactgttgagctgtaacaagttgtctcaggtgttcaatttcatgttctagttgctttgttttactggtttcacctgttctattaggtgttacatgctgttcatctgttacattgtcgatctgttcatggtgaacagctttgaatgcaccaaaaactcgtaaaagctctgatgtatctatcttttttacaccgttttcatctgtgcatatggacagttttccctttgatatgtaacggtgaacagttgttctacttttgtttgttagtcttgatgcttcactgatagatacaagagccataagaacctcagatccttccgtatttagccagtatgttctctagtgtggttcgttgtttttgcgtgagccatgagaacgaaccattgagatcatacttactttgcatgtcactcaaaaattttgcctcaaaactggtgagctgaatttttgcagttaaagcatcgtgtagtgtttttcttagtccgttatgtaggtaggaatctgatgtaatggttgttggtattttgtcaccattcatttttatctggttgttctcaagttcggttacgagatccatttgtctatctagttcaacttggaaaatcaacgtatcagtcgggcggcctcgcttatcaaccaccaatttcatattgctgtaagtgtttaaatctttacttattggtttcaaaacccattggttaagccttttaaactcatggtagttattttcaagcattaacatgaacttaaattcatcaaggctaatctctatatttgccttgtgagttttcttttgtgttagttcttttaataaccactcataaatcctcatagagtatttgttttcaaaagacttaacatgttccagattatattttatgaatttttttaactggaaaagataaggcaatatctcttcactaaaaactaattctaatttttcgcttgagaacttggcatagtttgtccactggaaaatctcaaagcctttaaccaaaggattcctgatttccacagttctcgtcatcagctctctggttgctttagctaatacaccataagcattttccctactgatgttcatcatctgagcgtattggttataagtgaacgataccgtccgttctttccttgtagggttttcaatcgtggggttgagtagtgccacacagcataaaattagcttggtttcatgctccgttaagtcatagcgactaatcgctagttcatttgctttgaaaacaactaattcagacatacatctcaattggtctaggtgattttaatcactataccaattgagatgggctagtcaatgataattactagtccttttcccgggtgatctgggtatctgtaaattctgctagacctttgctggaaaacttgtaaattctgctagaccctctgtaaattccgctagacctttgtgtgttttttttgtttatattcaagtggttataatttatagaataaagaaagaataaaaaaagataaaaagaatagatcccagccctgtgtataactcactactttagtcagttccgcagtattacaaaaggatgtcgcaaacgctgtttgctcctctacaaaacagaccttaaaaccctaaaggcttaagtagcaccctcgcaagctcgggcaaatcgctgaatattccttttgtctccgaccatcaggcacctgagtcgctgtctttttcgtgacattcagttcgctgcgctcacggctctggcagtgaatgggggtaaatggcactacaggcgccttttatggattcatgcaaggaaactacccataatacaagaaaagcccgtcacgggcttctcagggcgttttatggcgggtctgctatgtggtgctatctgactttttgctgttcagcagttcctgccctctgattttccagtctgaccacttcggattatcccgtgacaggtcattcagactggctaatgcacccagtaaggcagcggtatcatcaacaggcttacccgtcttactgtccctagtgcttggattctcaccaataaaaaacgcccggcggcaaccgagcgttctgaacaaatccagatggagttctgaggtcattactggatctatcaacaggagtccaagcgagctctcgaaccccagagtcccgctcagaagaactcgtcaagaaggcgatagaaggcgatgcgctgcgaatcgggagcggcgataccgtaaagcacgaggaagcggtcagcccattcgccgccaagctcttcagcaatatcacgggtagccaacgctatgtcctgatagcggtccgccacacccagccggccacagtcgatgaatccagaaaagcggccattttccaccatgatattcggcaagcaggcatcgccatgggtcacgacgagatcctcgccgtcgggcatgcgcgccttgagcctggcgaacagttcggctggcgcgagcccctgatgctcttcgtccagatcatcctgatcgacaagaccggcttccatccgagtacgtgctcgctcgatgcgatgtttcgcttggtggtcgaatgggcaggtagccggatcaagcgtatgcagccgccgcattgcatcagccatgatggatactttctcggcaggagcaaggtgagatgacaggagatcctgccccggcacttcgcccaatagcagccagtcccttcccgcttcagtgacaacgtcgagcacagctgcgcaaggaacgcccgtcgtggccagccacgatagccgcgctgcctcgtcctgcagttcattcagggcaccggacaggtcggtcttgacaaaaagaaccgggcgcccctgcgctgacagccggaacacggcggcatcagagcagccgattgtctgttgtgcccagtcatagccgaatagcctctccacccaagcggccggagaacctgcgtgcaatccatcttgttcaatcatgcgaaacgatcctcatcctgtctcttgatcagatcatgatcccctgcgccatcagatccttggcggcaagaaagccatccagtttactttgcagggcttcccaaccttaccagagggcgccccagctggcaattccgacgtc | Blue: pAR promoter  Green: yemGFP |
| pTargetF-fadD-YFP | tgacgactgacttaacgctcgttttagagctagaaatagcaagttaaaataaggctagtccgttatcaacttgaaaaagtggcaccgagtcggtgctttttttgaattctctagagtcgacctgcagaagcttagatctggttttaacgtctatcccaacgagattgaagatgtcgtcatgcagcatcctggcgtacaggaagtcgcggctgttggcgtaccttccggctccagtggtgaagcggtgaaaatcttcgtagtgaaaaaagatccatcgcttaccgaagagagcttagtgactttttgccgccgtcagctcacgggatacaaagtaccgaagctggtggagtttcgtgatgagttaccgaaatctaacgtcggaaaaattttgcgacgagaattacgtgacgaagcgcgcggcaaagtggacaataaagccggtggtagcactccctctaacggcggcggcgacgacggcagcggtggtagcaaaggtgaagaactgttcaccggcgttgtgccaattctggttgagctggatggtgacgtgaatggccacaaattttccgtgtctggtgaaggcgagggtgatgctacttatggcaaactgactctgaaactgatctgtaccaccggcaaactgcctgttccgtggccaactctggtcactactctgggttacggcgtgcagtgttttgcgcgttacccggatcacatgaaacagcatgacttcttcaaatctgccatgccggaaggctatgtccaagaacgtacgatctttttcaaggacgacggcaactataaaacccgtgccgaagttaaattcgagggtgacaccctggttaaccgcatcgaactgaaaggcattgacttcaaagaggacggcaacattctgggtcacaagctggaatacaactacaactcccacaacgtttacattactgctgacaagcagaaaaacggcatcaaagcaaacttcaagatccgtcacaacattgaagatggtggcgtacagctggcagatcactaccagcagaacactccaatcggtgatggcccagtactgctgccagataaccattacctgtcctaccagagcaaactgtctaaagacccgaacgaaaaacgtgaccacatggtactgctggaatttgttaccgcggcaggcattaccctgggtatggacgaactgtataaataatgagcgttaagtcagtcgtcagacgccggttaatccggcgttttttttgacgcccactaaagagaaaacaatttgaattaccaaatgattaccacggacgatgcgctggcttctttgtgtgaagccgtccgtgcctttccggcgatagccctggatactgaatttgttcgtacgcgcacttattacccgcagctggggttctcgagttcatgtgcagctccatcagcaaaaggggatgataagtttatcaccaccgactatttgcaacagtgccgttgatcgtgctatgatcgactgatgtcatcagcggtggagtgcaatgtcatgagggaagcggtgatcgccgaagtatcgactcaactatcagaggtagttggcgtcatcgagcgccatctcgaaccgacgttgctggccgtacatttgtacggctccgcagtggatggcggcctgaagccacacagtgatattgatttgctggttacggtgaccgtaaggcttgatgaaacaacgcggcgagctttgatcaacgaccttttggaaacttcggcttcccctggagagagcgagattctccgcgctgtagaagtcaccattgttgtgcacgacgacatcattccgtggcgttatccagctaagcgcgaactgcaatttggagaatggcagcgcaatgacattcttgcaggtatcttcgagccagccacgatcgacattgatctggctatcttgctgacaaaagcaagagaacatagcgttgccttggtaggtccagcggcggaggaactctttgatccggttcctgaacaggatctatttgaggcgctaaatgaaaccttaacgctatggaactcgccgcccgactgggctggcgatgagcgaaatgtagtgcttacgttgtcccgcatttggtacagcgcagtaaccggcaaaatcgcgccgaaggatgtcgctgccgactgggcaatggagcgcctgccggcccagtatcagcccgtcatacttgaagctagacaggcttatcttggacaagaagaagatcgcttggcctcgcgcgcagatcagttggaagaatttgtccactacgtgaaaggcgagatcaccaaggtagtcggcaaataagatgccgctcgccagtcgattggctgagctcatgaagttcctattccgaagttccgcgaacgcgtaaaggatctaggtgaagatcctttttgataatctcatgaccaaaatcccttaacgtgagttttcgttccactgagcgtcagaccccgtagaaaagatcaaaggatcttcttgagatcctttttttctgcgcgtaatctgctgcttgcaaacaaaaaaaccaccgctaccagcggtggtttgtttgccggatcaagagctaccaactctttttccgaaggtaactggcttcagcagagcgcagataccaaatactgtccttctagtgtagccgtagttaggccaccacttcaagaactctgtagcaccgcctacatacctcgctctgctaatcctgttaccagtggctgctgccagtggcgataagtcgtgtcttaccgggttggactcaagacgatagttaccggataaggcgcagcggtcgggctgaacggggggttcgtgcacacagcccagcttggagcgaacgacctacaccgaactgagatacctacagcgtgagctatgagaaagcgccacgcttcccgaagggagaaaggcggacaggtatccggtaagcggcagggtcggaacaggagagcgcacgagggagcttccagggggaaacgcctggtatctttatagtcctgtcgggtttcgccacctctgacttgagcgtcgatttttgtgatgctcgtcaggggggcggagcctatggaaaaacgccagcaacgcggcctttttacggttcctggccttttgctggccttttgctcacatgttctttcctgcgttatcccctgattctgtggataaccgtattaccgcctttgagtgagctgataccgctcgccgcagccgaacgaccgagcgcagcgagtcagtgagcgaggaagcggaagagcgcctgatgcggtattttctccttacgcatctgtgcggtatttcacaccgcatatgctggatccttgacagctagctcagtcctaggtataatactagt | Blue: *fadD* N20,  Red: *fadD* homology arms,  Purple: Glycine-Serine rich linker; Yellow: YFP |
| NCM3722 φ(fadD-YFP) *fadD* genome region | ttgcttgtttttaaagaaaaagaaacagcggctggtccgctgtttctgcattcttacggtaaagataaaaataaatagtgacgcgcttcgcaaccttttcgttgggtaattatcaagctggtatgatgagttaatattatgttaacggcatgtatatcatttggggttgcgatgacgacgaacacgcattttagaggtgaagaattgaagaaggtttggcttaaccgttatcccgcggacgttccgacggagatcaaccctgaccgttatcaatctctggtagatatgtttgagcagtcggtcgcgcgctacgccgatcaacctgcgtttgtgaatatgggggaggtaatgaccttccgcaagctggaagaacgcagtcgcgcgtttgccgcttatttgcaacaagggttggggctgaagaaaggcgatcgcgttgcgttgatgatgcctaatttattgcaatatccggtggcgctgtttggcattttgcgtgccgggatgatcgtcgtaaacgttaacccgttgtataccccgcgtgagcttgagcatcagcttaacgatagcggcgcatcggcgattgttatcgtgtctaactttgctcacacactggaaaaagtggttgataaaaccgccgttcagcacgtaattctgacccgtatgggcgatcagctatctacggcaaaaggcacggtagtcaatttcgttgttaaatacatcaagcgtttggtgccgaaataccatctgccagatgccatttcatttcgtagcgcactgcataacggctaccggatgcagtacgtcaaacccgaactggtgccggaagatttagcttttctgcaatacaccggcggcaccactggtgtggcgaaaggcgcgatgctgactcaccgcaatatgctggcgaacctggaacaggttaacgcgacctatggtccgctgttgcatccgggcaaagagctggtggtgacggcgctgccgctgtatcacatttttgccctgaccattaactgcctgctgtttatcgaactgggtgggcagaacctgcttatcactaacccgcgcgatattccagggttggtaaaagagttagcgaaatatccgtttaccgctatcacgggcgttaacaccttgttcaatgcgttgctgaacaataaagagttccagcagctggatttctccagtctgcatctttccgcaggcggtgggatgccagtgcagcaagtggtggcagagcgttgggtgaaactgaccggacagtatctgctggaaggctatggccttaccgagtgtgcgccgctggtcagcgttaacccatatgatattgattatcatagtggtagcatcggtttgccggtgccgtcgacggaagccaaactggtggatgatgatgataatgaagtaccaccaggtcaaccgggtgagctttgtgtcaaaggaccgcaggtgatgctgggttactggcagcgtcccgatgctaccgatgaaatcatcaaaaatggctggttacacaccggcgacatcgcggtaatggatgaagaaggattcctgcgcattgtcgatcgtaaaaaagacatgattctggtttccggttttaacgtctatcccaacgagattgaagatgtcgtcatgcagcatcctggcgtacaggaagtcgcggctgttggcgtaccttccggctccagtggtgaagcggtgaaaatcttcgtagtgaaaaaagatccatcgcttaccgaagagtcactggtgactttttgccgccgtcagctcacgggatacaaagtaccgaagctggtggagtttcgtgatgagttaccgaaatctaacgtcggaaaaattttgcgacgagaattacgtgacgaagcgcgcggcaaagtggacaataaagccggtggtagcactccctctaacggcggcggcgacgacggcagcggtggtagcaaaggtgaagaactgttcaccggcgttgtgccaattctggttgagctggatggtgacgtgaatggccacaaattttccgtgtctggtgaaggcgagggtgatgctacttatggcaaactgactctgaaactgatctgtaccaccggcaaactgcctgttccgtggccaactctggtcactactctgggttacggcgtgcagtgttttgcgcgttacccggatcacatgaaacagcatgacttcttcaaatctgccatgccggaaggctatgtccaagaacgtacgatctttttcaaggacgacggcaactataaaacccgtgccgaagttaaattcgagggtgacaccctggttaaccgcatcgaactgaaaggcattgacttcaaagaggacggcaacattctgggtcacaagctggaatacaactacaactcccacaacgtttacattactgctgacaagcagaaaaacggcatcaaagcaaacttcaagatccgtcacaacattgaagatggtggcgtacagctggcagatcactaccagcagaacactccaatcggtgatggcccagtactgctgccagataaccattacctgtcctaccagagcaaactgtctaaagacccgaacgaaaaacgtgaccacatggtactgctggaatttgttaccgcggcaggcattaccctgggtatggacgaactgtataaataatgagcgttaagtcagtcgtcagacgccggttaatccggcgttttttttgacgcccactaaagagaaaacaat | Red: *fadD* CDS  Purple: Glycine-Serine rich linker; Yellow: YFP |
